# Mutation Patterns in the Human Genome: More Variable Than Expected

**DOI:** 10.1371/journal.pbio.1000028

**Published:** 2009-02-03

**Authors:** Laurent Duret

## Abstract

Why are some genomic positions more mutable than others? The identification of cryptic mutation hotspots in the human genome indicates that the determinants of mutation rates are more complex than anticipated.

The development, survival, and reproduction of an organism depend on the genetic information that is carried in its genome, yet the transmission of genetic information is not perfectly accurate: new mutations occur at each generation. These mutations are the primary cause of the genetic diversity on which natural selection can operate, and hence are the sine qua non of evolution. A better knowledge of mutation processes is crucial for investigating the causes of genetic diseases or cancer and for understanding evolutionary processes. This knowledge is also important for different practical reasons. First, comparative sequence analysis is widely used to find functional elements within genomes. The basic principle of this approach is that functional elements are affected by natural selection, and hence can be recognized because they evolve either slower or faster than expected given the local mutation rate. Hence, to be able to annotate genomic sequences, it is necessary to have a good knowledge of the underlying pattern of mutation. Moreover, this knowledge is also essential for ensuring the accuracy of the methods that analyze sequence divergence to determine the phylogeny of species or the demographical history of populations. Finally, the study of mutational processes also provides valuable information about genome function in processes such as replication, repair, transcription, and recombination. During the last few years, several important factors affecting mutation rates have been uncovered. However, a paper in this issue of *PLoS Biology* [1] reveals an unexpected additional layer of complexity in the determinants of mutation rates.

A priori, nucleotide mutation rates are expected to depend upon three factors [2]: (i) the intrinsic stability of nucleotides and their sensitivity to mutagenic agents; (ii) the fidelity of DNA replication; and (iii) the efficiency of the DNA repair machinery. The analysis of variations in mutation rate across genomes can shed light on the relative contribution of these different factors and on the genomic features that affect mutation rates. In mammals, current knowledge of mutation processes derives essentially from the analysis of a limited number of germ-line mutations responsible for human genetic diseases [3] and from phylogenetic studies. This latter approach consists of comparing homologous sequences (between species or within populations) to estimate the number and kinds of changes that occurred since their divergence. At neutral sites—i.e., sites where the impact of natural selection is presumed to be null or very limited (pseudogenes, defective transposable elements, noncoding sequences, synonymous codon positions)—substitution rate is expected to be equal to the mutation rate [4]. This approach suffers from several limitations (see below), but thanks to the accumulation of genome sequences and polymorphism data, it has provided indirect estimates of genome-wide mutation patterns.

## Large-Scale Variations in the Rate and Patterns of Neutral Sequence Evolution

Phylogenetic analyses show that in mammals, neutral rates of sequence evolution (measured in number of base changes per site and per year) vary at different scales. First, substitution rates vary between species. Notably, species with short generation time generally evolve faster, presumably because they experience more rounds of germ-cell divisions (and hence more DNA replication errors) during a given unit of time [5]. If most mutations are due to DNA replication errors, then mutation rates are expected to be higher in males than in females, owing to the greater number of cell divisions per generation in the male germ-line. In agreement with that prediction, in apes, substitution rate is two times higher on the Y chromosome than on the X, whereas autosomes show intermediate values (the three classes of chromosomes spend on average—over generations—respectively 100%, 33%, and 50% of their time in the male germ-line) [6,7]. The strength of this male mutation bias in different mammalian species appears to be correlated to the number of male germ-cell divisions [8]. Within autosomes, there are substantial variations in neutral substitution rates at the megabase scale [7,9–11], but it is not clear to what extent these variations reflect mutational processes or are only the consequence of biased gene conversion (i.e., the biased repair of mismatches occurring in heteroduplex DNA during meiotic recombination), a neutral process that affects the probability of fixation of mutations [12]. Patterns of neutral substitution vary also at the gene scale. Notably, mammalian genomes show an excess of A→G transitions over T→C transitions, specifically in transcribed regions [13]. This may be a consequence of transcription-coupled repair [13]. Finally, it is well known that some short sequence motifs (such as minisatellite or microsatellite repeats, typically less than 100 bp long) are prone to DNA replication errors [14].

## Fine-Scale Variations in Substitution Rates: Neighbor-Dependant Mutational Processes

In mammals, the most dramatic variation in mutation rate is observed at the dinucleotide scale: a cytosine followed by a guanine is about 10 times more mutable than a cytosine in any other dinucleotide context [15,16]. Mutations of cytosines in CG dinucleotide (conventionally noted CpG, “p” standing for the phosphate between the two bases) are responsible for one third of disease-causing germ-line mutations in humans [17]. CpGs are the target of cytosine methylases in mammals, and their hypermutability is the consequence of the spontaneous deamination of methylated cytosines into thymines [18]. Compared to other substitutions, CpG substitution rates show weaker male mutation bias, which is consistent with the fact that the majority of mutations at CpG sites are not due to DNA replication errors [7]. The rate of substitution at CpG sites is strongly negatively correlated to the regional GC-content [12,19,20]. The influence of GC-content on CpG substitution appears to be very local (less than 2 kb) [20]. This observation is linked to the fact that cytosine deamination occurs essentially in single-stranded DNA [21]. Hence, the rate of CpG mutation is expected to depend on the rate of DNA melting, which is affected by the local base composition—GC-rich DNA fragment being more stable [21].

Although less dramatic, 2- to 3-fold variations in substitution rates are observed in other dinucleotide contexts [16,22]. These variations are poorly understood, but are probably the consequence of context-dependant DNA-replication errors [22]. Finally, substitution rates vary also at the base pair scale: in primates, substitution rates at G:C base pairs (excluding CpG sites) are 25% to 85% higher than at A:T base pairs [11,12], possibly because cytosine is intrinsically more mutable than other bases [23].

## Mutagenic Effects of Heterozygosity?

A recent study suggested that the probability of mutation at a given site might be affected by the presence of polymorphic sites in its vicinity [24]. By comparing pairs of closely related species in different eukaryotic taxa, the authors showed that the occurrence of an insertion or deletion (indel) in a given species is associated with an excess of single-nucleotide changes in the flanking region (less than 150 bp) in the same species [24]. Selection is unlikely to explain this clustering of changes in a given lineage because selection is a priori expected to affect both species equally [24]. The authors proposed that the heterozygosity for an indel might promote mutations in surrounding sequences, possibly because the repair of indel mismatches in heteroduplex DNA during meiotic recombination might be error-prone [24]. Along the same lines, it has been proposed that the repair of hypermutable CpG sites might be the cause of the high substitution rate observed in flanking non-CpG sites [25]. The hypothesis that sequence polymorphism is mutagenic remains to be demonstrated, but if confirmed, it raises the intriguing possibility that the rate of mutation in sexual species might also be affected by population parameters, such as effective population size and migration.

## Cryptic Mutational Hotspots

Now, research by Hodgkinson and colleagues published in this issue of *PLoS Biology* [1] reveals a new level of variation in mutation rate, which is not associated with any obvious sequence feature. The authors investigated the pattern of single nucleotide polymorphism (SNP) in human populations, at sites that are known to be polymorphic in chimpanzee. If some sites are more prone to mutations than others, then one expects to find an excess of sites that are polymorphic in both species (coincident SNPs). And indeed, the observed number of coincident SNPs is three times higher than the number expected under the null hypothesis that SNPs are randomly distributed in the two genomes. Even after accounting for the effects of the hypermutability of CpGs and other neighbor-dependant mutational processes, the authors still find a 1.76-fold excess of coincident SNPs. Interestingly, this excess is essentially due to the same SNP (i.e., the same pair of alleles) being present in both species.

Such SNPs could potentially correspond to ancestral polymorphism, present in the last common ancestor of human and chimpanzee, and preserved in both lineages. However, comparison with macaque revealed the same excess of coincident SNPs, whereas very few polymorphisms are expected to be shared between human and macaque given their divergence time. Moreover, the hypothesis of ancestral polymorphism predicts that all categories of SNPs should show the same frequency of coincident SNPs, whereas they observed a particularly striking excess specifically for A/T coincident SNPs. Might the excess of coincident SNPs be the consequence of selection? Positive selection leads to the rapid fixation of advantageous mutations, and hence is not expected to lead to an excess of coincident SNPs. Negative selection reduces polymorphism at functional sites and hence might lead to a clustering of SNPs in nonfunctional regions. However, the data show no tendency for SNPs to cluster (which is not surprising given that 98% of the analyzed data set consists of intergenic or intronic regions—where only a very small fraction of sites are expected to be under selective pressure). If the excess of coincident SNPs is due neither to selection nor to ancestral polymorphism, then it must reflect an excess of convergent mutations, occurring independently at the same sites in both species. This phenomenon is quantitatively important: indeed, the level of variation in mutation rates that is necessary to account for the observed number of coincident SNPs is similar, or even higher, than the level of variation that is due to CpG hypermutability [1].

To investigate the sequence features that might be responsible for these mutational hotspots, the authors analyzed the sequence composition in regions flanking coincident SNPs. Although they noticed that the frequency of particular triplet oligonucleotides was significantly different in the 100 bp surrounding coincident SNPs compared to other SNPs, they were unable to identify any clear sequence motif that could explain a substantial fraction of these hotspots [1].

## Direct Evaluation of Mutation Patterns in Mammals

The discovery of cryptic mutational hotspots in the human genome [1] illustrates how limited our knowledge of the determinants of mutation rates remains. Thanks to large-scale sequencing projects, genome-wide substitution patterns can be measured in different mammalian taxa [5]. However, there is now clear evidence that in mammals, biased gene conversion has a strong impact on genome-wide substitution patterns [12]. Hence, even at neutral sites, substitution rates do not provide a perfect estimator of mutation rates. As mentioned previously, a precise knowledge of genome-wide mutation patterns is crucial for many issues in genetics or evolutionary biology. Notably, to be able to detect functional elements within genomes, it is essential to tease apart the relative contribution of the three determinants of sequence evolution: mutation, biased gene conversion, and selection. This will require a direct quantification of mutation patterns.

Thanks to the new technologies, it is now feasible to directly measure mutation rates by sequencing. This approach has first been used in species with relatively small genomes compared to mammals (yeast, drosophila, nematode) [26–28]. Recently, direct whole-genome sequencing was used to identify somatic mutations in a human tumor [29]. Hopefully, it will soon be possible to directly measure germ-line mutation rates in humans by sequencing the genomes of a mother, a father, and their child.

## Abbreviations

SNP: single nucleotide polymorphism

## References

[pbio-1000028-b001] Hodgkinson A, Ladoukakis E, Eyre-Walker A (2009). Cryptic variation in the human mutation rate. PLoS Biol.

[pbio-1000028-b002] Baer CF, Miyamoto MM, Denver DR (2007). Mutation rate variation in multicellular eukaryotes: Causes and consequences. Nat Rev Genet.

[pbio-1000028-b003] Kondrashov AS (2003). Direct estimates of human per nucleotide mutation rates at 20 loci causing Mendelian diseases. Hum Mutat.

[pbio-1000028-b004] Kimura M (1968). Evolutionary rate at the molecular level. Nature.

[pbio-1000028-b005] Nikolaev SI, Montoya-Burgos JI, Popadin K, Parand L, Margulies EH (2007). Life-history traits drive the evolutionary rates of mammalian coding and noncoding genomic elements. Proc Natl Acad Sci U S A.

[pbio-1000028-b006] Makova KD, Li WH (2002). Strong male-driven evolution of DNA sequences in humans and apes. Nature.

[pbio-1000028-b007] Chimpanzee Sequencing and Analysis Consortium (2005). Initial sequence of the chimpanzee genome and comparison with the human genome. Nature.

[pbio-1000028-b008] Goetting-Minesky MP, Makova KD (2006). Mammalian male mutation bias: Impacts of generation time and regional variation in substitution rates. J Mol Evol.

[pbio-1000028-b009] Matassi G, Sharp PM, Gautier C (1999). Chromosomal location effects on gene sequence evolution in mammals. Curr Biol.

[pbio-1000028-b010] Hardison RC, Roskin KM, Yang S, Diekhans M, Kent WJ (2003). Covariation in frequencies of substitution, deletion, transposition, and recombination during eutherian evolution. Genome Res.

[pbio-1000028-b011] Arndt PF, Hwa T, Petrov DA (2005). Substantial regional variation in substitution rates in the human genome: Importance of GC content, gene density, and telomere-specific effects. J Mol Evol.

[pbio-1000028-b012] Duret L, Arndt PF (2008). The impact of recombination on nucleotide substitutions in the human genome. PLoS Genet.

[pbio-1000028-b013] Green P, Ewing B, Miller W, Thomas PJ, Green ED (2003). Transcription-associated mutational asymmetry in mammalian evolution. Nat Genet.

[pbio-1000028-b014] Jeffreys AJ, Royle NJ, Wilson V, Wong Z (1988). Spontaneous mutation rates to new length alleles at tandem-repetitive hypervariable loci in human DNA. Nature.

[pbio-1000028-b015] Bird AP (1980). DNA methylation and the frequency of CpG in animal DNA. Nucleic Acids Res.

[pbio-1000028-b016] Hess ST, Blake JD, Blake RD (1994). Wide variations in neighbor-dependent substitution rates. J Mol Biol.

[pbio-1000028-b017] Cooper DN, Krawczak M (1990). The mutational spectrum of single base-pair substitutions causing human genetic disease: patterns and predictions. Hum Genet.

[pbio-1000028-b018] Coulondre C, Miller JH, Farabaugh PJ, Gilbert W (1978). Molecular basis of base substitution hotspots in Escherichia coli. Nature.

[pbio-1000028-b019] Fryxell KJ, Moon WJ (2005). CpG mutation rates in the human genome are highly dependent on local GC content. Mol Biol Evol.

[pbio-1000028-b020] Elango N, Kim S-H, Vigoda E, Yi SV (2008). Mutations of different molecular origins exhibit contrasting patterns of regional substitution rate variation. PLoS Comput Biol.

[pbio-1000028-b021] Frederico LA, Kunkel TA, Shaw BR (1993). Cytosine deamination in mismatched base pairs. Biochemistry.

[pbio-1000028-b022] Hwang DG, Green P (2004). Bayesian Markov chain Monte Carlo sequence analysis reveals varying neutral substitution patterns in mammalian evolution. Proc Natl Acad Sci U S A.

[pbio-1000028-b023] Birdsell JA (2002). Integrating genomics, bioinformatics, and classical genetics to study the effects of recombination on genome evolution. Mol Biol Evol.

[pbio-1000028-b024] Tian D, Wang Q, Zhang P, Araki H, Yang S (2008). Single-nucleotide mutation rate increases close to insertions/deletions in eukaryotes. Nature.

[pbio-1000028-b025] Walser JC, Ponger L, Furano AV (2008). CpG dinucleotides and the mutation rate of non-CpG DNA. Genome Res.

[pbio-1000028-b026] Denver DR, Morris K, Lynch M, Thomas WK (2004). High mutation rate and predominance of insertions in the Caenorhabditis elegans nuclear genome. Nature.

[pbio-1000028-b027] Haag-Liautard C, Coffey N, Houle D, Lynch M, Charlesworth B (2008). Direct estimation of the mitochondrial DNA mutation rate in Drosophila melanogaster. PLoS Biol.

[pbio-1000028-b028] Lynch M, Sung W, Morris K, Coffey N, Landry CR (2008). A genome-wide view of the spectrum of spontaneous mutations in yeast. Proc Natl Acad Sci U S A.

[pbio-1000028-b029] Ley TJ, Mardis ER, Ding L, Fulton B, McLellan MD (2008). DNA sequencing of a cytogenetically normal acute myeloid leukaemia genome. Nature.

